# Genome-wide association study of shared components of reading disability and language impairment

**DOI:** 10.1111/gbb.12085

**Published:** 2013-09-09

**Authors:** J D Eicher, N R Powers, L L Miller, N Akshoomoff, D G Amaral, C S Bloss, O Libiger, N J Schork, B F Darst, B J Casey, L Chang, T Ernst, J Frazier, W E Kaufmann, B Keating, T Kenet, D Kennedy, S Mostofsky, S S Murray, E R Sowell, H Bartsch, J M Kuperman, T T Brown, D J Hagler, A M Dale, T L Jernigan, B St Pourcain, G Davey Smith, S M Ring, J R Gruen

**Affiliations:** † Department of Genetics, Yale UniversityNew Haven, CT, USA; ‡ MRC Integrative Epidemiology Unit (IEU), School of Social and Community Medicine, University of BristolBristol, UK; § Center for Human Development, University of California at San DiegoLa Jolla, CA, USA; ¶ Department of Psychiatry, University of California at San DiegoLa Jolla, CA, USA; ** Department of Psychiatry and Behavioral Sciences, University of CaliforniaDavis, CA, USA; †† Scripps Genomic Medicine, Scripps Translational Science Institute and Scripps HealthLa Jolla, CA, USA; ‡‡ Sackler Institute for Developmental Psychobiology, Weil Cornell Medical CollegeNew York, NY, USA; §§ Department of Medicine, University of Hawaii and Queen's Medical CenterHonolulu, HI, USA; ¶¶ Department of Psychiatry, University of Massachusetts Medical SchoolBoston, MA, USA; *** Kennedy Krieger InstituteBaltimore, MD, USA; ††† Department of Neurology, Children's Hospital Boston, Harvard Medical SchoolBoston, MA, USA; ‡‡‡ Department of Neurology and Athinoula A. Martinos Center for Biomedical Imaging, Massachusetts General HospitalCharlestown, MA, USA; §§§ Department of Pediatrics, University of Southern CaliforniaLos Angeles, CA, USA; ¶¶¶ Developmental Cognitive Neuroimaging Laboratory, Children's HospitalLos Angeles, CA, USA; **** Multimodal Imaging Laboratory, University of California at San DiegoLa Jolla, CA, USA; †††† Department of Neurosciences, University of California at San DiegoLa Jolla, CA, USA; ‡‡‡‡ Department of Radiology, University of California at San DiegoLa Jolla, CA, USA; §§§§ Department of Cognitive Science, University of California at San DiegoLa Jolla, CA, USA; ¶¶¶¶ School of Oral and Dental Sciences, University of BristolBristol, UK; ***** School of Experimental Psychology, University of BristolBristol, UK; ††††† Departments of Pediatrics and Investigative Medicine, Yale University School of MedicineNew Haven, CT, USA

**Keywords:** ALSPAC, dyslexia GWAS, language impairment, PING, reading disability, ZNF385D

## Abstract

Written and verbal languages are neurobehavioral traits vital to the development of communication skills. Unfortunately, disorders involving these traits—specifically reading disability (RD) and language impairment (LI)—are common and prevent affected individuals from developing adequate communication skills, leaving them at risk for adverse academic, socioeconomic and psychiatric outcomes. Both RD and LI are complex traits that frequently co-occur, leading us to hypothesize that these disorders share genetic etiologies. To test this, we performed a genome-wide association study on individuals affected with both RD and LI in the Avon Longitudinal Study of Parents and Children. The strongest associations were seen with markers in *ZNF385D* (OR = 1.81, *P* = 5.45 × 10^−7^) and *COL4A2* (OR = 1.71, *P* = 7.59 × 10^−7^). Markers within *NDST4* showed the strongest associations with LI individually (OR = 1.827, *P* = 1.40 × 10^−7^). We replicated association of *ZNF385D* using receptive vocabulary measures in the Pediatric Imaging Neurocognitive Genetics study (*P* = 0.00245). We then used diffusion tensor imaging fiber tract volume data on 16 fiber tracts to examine the implications of replicated markers. *ZNF385D* was a predictor of overall fiber tract volumes in both hemispheres, as well as global brain volume. Here, we present evidence for *ZNF385D* as a candidate gene for RD and LI. The implication of transcription factor *ZNF385D* in RD and LI underscores the importance of transcriptional regulation in the development of higher order neurocognitive traits. Further study is necessary to discern target genes of *ZNF385D* and how it functions within neural development of fluent language.

The development of reading and verbal language skills through early childhood and into adolescence is vital to a child's academic performance, self-perception of cognitive abilities and development of sociability. Reading disability (RD) and language impairment (LI) are two common language-based learning disabilities with prevalence estimates of 5–17% and 5–8%, respectively (Pennington & Bishop 2009; Peterson & Pennington 2012). RD and LI are characterized by unexplained difficulties in written and verbal language, respectively, despite adequate intelligence, educational and socioeconomic opportunity (Pennington & Bishop 2009; Peterson & Pennington 2012). RD and LI have lifelong detrimental effects on communication and language skills, particularly without early intervention. RD and LI are frequently comorbid; e.g. children diagnosed with LI are more likely to develop RD later in childhood (Pennington 2006). Additionally, children with RD and/or LI exhibit deficits in many of the same neurocognitive domains, including phonological processing, comprehension, fluency and phonological short-term memory (Catts *et al.* 2005; Gathercole & Baddeley 1990; Pennington 2006; Pennington & Bishop 2009; Wise *et al.* 2007).

The relatedness between RD and LI goes deeper than similarity in clinical presentation. RD and LI share numerous risk factors and associated genes, as both are complex disorders with substantial genetic contributors (Pennington & Bishop 2009; Scerri & Schulte-Korne 2010). Linkage, candidate gene association and rare variant studies have identified genes that contribute to RD and/or LI (Graham & Fisher 2013; Newbury *et al.* 2009, 2011; Pinel *et al.* 2012; Rice *et al.* 2009; Scerri *et al.* 2011). Some of these risk genes, including *DCDC2*, *KIAA0319*, *FOXP2*, *CNTNAP2* and *CMIP*, contribute to both RD and LI (Newbury *et al.* 2011; Peter *et al.* 2011; Powers *et al.* 2013; Scerri *et al.* 2011; Wilcke *et al.* 2011). These studies suggest that RD and LI share certain risk genes that influence core language processes. However, genome-wide association studies (GWAS) on reading and language are limited. Recently, Luciano *et al.* (2013) completed a GWAS on quantitative performance on reading- and language-related measures. The strongest associations were seen between *ABCC13* and nonword repetition. These analyses identified novel genes and loci for performance on written and verbal language tasks, but do not address disorder states (i.e. RD or LI) nor the common comorbidity of RD and LI.

Neuroimaging studies of written and verbal language have identified various brain regions and measures important for fluent language and altered in impaired individuals (Shaywitz & Shaywitz 2008; Vandermosten *et al.* 2012). Some argue that these imaging differences may represent a mediatory step between genetic risk variants and the ultimate clinical phenotype (Eicher & Gruen 2013). Thus, recent studies have used these neuroimaging measures as endophenotypes in their analyses. These imaging-genetic studies have associated RD and LI risk genes—including *FOXP2*, *CNTNAP2*, *KIAA0319*, *DCDC2* and *C2orf3*—with various brain imaging phenotypes—including brain activation patterns, white and grey matter volumes and fiber tract volumes (Cope *et al.* 2012; Darki *et al.* 2012; Eicher & Gruen 2013; Liegeois *et al.* 2003; Pinel *et al.* 2012; Scott-Van Zeeland *et al.* 2010; Scerri *et al.* 2012; Tan *et al.* 2010; Wilcke *et al.* 2011).

The goal of this investigation is to identify novel genes that contribute to the overlap of RD and LI by performing a GWAS on subjects with both RD and LI in an extensively phenotyped birth cohort: the Avon Longitudinal Study of Parents and Children (ALSPAC). The large number of neurocognitive assessments in the ALSPAC allows for the simultaneous analysis of RD and LI. By doing so, we aim to identify new genes that contribute to both RD and LI. We then replicate our results in the Pediatric Imaging Neurocognition Genetics (PING) study using oral reading and receptive vocabulary measures. For replicated markers, we test for associations with fiber tract volumes previously implicated in language.

## Materials and methods

### Avon Longitudinal Study of Parents and Children

Subject recruitment and collection of phenotype and genetic data for the ALSPAC cohort were completed by the ALSPAC team. The ALSPAC is a prospective population-based, birth cohort based on the Avon region of the UK. It consists mainly of children of northern European descent, born in 1991 and 1992. Children were recruited before birth; recruitment of their pregnant mothers resulted in a total of 15 458 fetuses, of whom 14 701 were alive at 1 year of age. Details regarding the participants, recruitment and study methodologies are described in detail elsewhere (http://www.bristol.ac.uk/alspac) (Boyd *et al.* 2012; Golding *et al.* 2001). The children of the ALSPAC have been extensively phenotyped from before birth to early adulthood. Ethical approval was obtained from the ALSPAC Ethics and Law Committee, Local UK Research Ethics Committees, and the Yale Human Investigation Committee.

### Reading and language measures

The reading, language and cognitive measures used for this study were collected at ages 7, 8 and 9 years. Subjects with IQ ≤ 75 on the Wechsler Intelligence Scale for Children (WISC-III) Total IQ, completed at age 8 years, were excluded from the presented analyses (Wechsler *et al.* 1992). Reading measures in the ALSPAC include a phoneme deletion task at age 7, single-word reading at ages 7 and 9 years, single nonword reading at age 9 years, and reading passage comprehension at age 9 years. The phoneme deletion task measures phoneme awareness, widely considered to be a core deficit in both RD and LI (Pennington 2006; Pennington & Bishop 2009). For the phoneme deletion task, also known as the Auditory Analysis Test, the child listens to a word spoken aloud, and is then asked to remove a specific phoneme from that word to make a new word (Rosner & Simon 1971). Single-word reading was assessed at age 7 using the reading subtest of the Wechsler Objective Reading Dimensions (WORD). At age 9, single-word and nonword reading were assessed by asking the child to read 10 real words and 10 nonwords aloud from a subset of a larger list of words and nonwords taken from research conducted by Terezinha Nunes and colleagues (Rust *et al.* 1993). Reading comprehension scores were ascertained at age 9, using the Neale Analysis of Reading Ability (NARA-II) (Neale 1997). Two additional language measures, nonword repetition and verbal comprehension tasks, were collected during clinical interviews at age 8 years. An adaptation of the Nonword Repetition Task (NWR), in which subjects repeated recordings of nonwords, was used to assess short-term phonological memory and processing (Gathercole & Baddeley 1996). Children also completed the Wechsler Objective Language Dimensions (WOLD) verbal comprehension task, where they answered questions about a paragraph read aloud by an examiner describing a presented picture (Wechsler 1996). *z*-Scores were calculated for each subject on each individual measure.

### Case definitions

**Figure 1:**
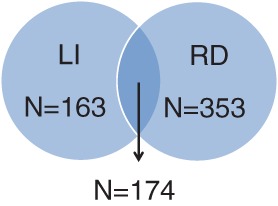
**Number of RD and LI cases in the ALSPAC cohort following the case definitions in Table 1.** There were 174 subjects with comorbid RD and LI. There were 163 subjects with LI without comorbid RD, and 353 subjects with RD without comorbid LI.

We aimed to capture persistently poor performers in various reading and verbal language domains as RD and LI cases in our case definitions (Table 1). Therefore, we defined RD cases as having a *z*-score less than or equal to −1 on at least 3 of the 5 following tasks: single-word reading at age 7 years, phoneme deletion at age 7 years, single-word reading at age 9 years, nonword reading at age 9 years, and reading comprehension at age 9 years. There were 527 subjects defined as RD cases. We defined LI cases as having a *z*-score less than or equal to −1 on at least 2 of the 3 following tasks: phoneme deletion at age 7 years, verbal comprehension at age 8 years, and nonword repetition at age 8 years. There were 337 subjects defined as LI cases. As phoneme awareness is important in both RD and LI, we chose to include it as a part of the case definition for both RD and LI to reflect clinical presentation. There were 174 individuals affected with both RD and LI, with a male to female ratio of 1.7:1. In the further characterization of observed associations, we created subsets of cases with no comorbidity. There were 163 LI cases excluding those with comorbid RD, and 353 RD cases excluding those with comorbid LI (Fig. 1). For all analyses, controls were defined as ALSPAC subjects of European ancestry who completed all the necessary neurobehavioral assessments but did not meet the criteria for case status.

**Table 1:** Reading and language measures used to define RD and LI cases

RD (*n* = 527)*	LI (*n* = 337)†
Phoneme deletion age 7 years	Phoneme deletion age 7 years
Single-word reading age 7 years	Verbal comprehension age 8 years
Single-word reading age 9 years	Nonword repetition age 8 years
Nonword reading age 9 years	
Reading comprehension age 9 years	

* RD cases had a *z*-score of less than or equal to −1 on at least 3 of the 5 reading measures.

† LI cases had a *z*-score of less than or equal to −1 on at least 2 of the 3 language measures.

### Genotyping and analysis

Subjects were genotyped on Illumina HumanHap 550 bead arrays (San Diego, CA, USA). Subjects were excluded if the percentage of missing genotypes was greater than 2% (*n* = 6). To prevent possible population stratification, only subjects of European ancestry were included. In our primary analysis of RD and LI individuals, there were 174 cases and 4117 controls. There were a total of 500 527 single nucleotide polymorphisms (SNPs) genotyped before quality assessment and quality control. Markers were removed if Hardy–Weinberg equilibrium *P* ≤ 0.0001 (*n* = 93) or if missingness was greater than 10% (*n* = 19). All markers had a minor allele frequency greater than 0.01. All genetic analyses were performed using logistic regression in plink v1.07 (Purcell *et al.* 2007). To correct for multiple testing, we set a Bonferroni corrected threshold of *α* = 1.00 × 10^−7^ = 0.05/500 000 markers tested.

Following our initial analyses examining cases with both RD and LI, we further examined RD and LI case definitions individually (i.e. LI excluding those with comorbid RD, and RD excluding those with comorbid LI). These analyses were completed to determine whether a single disorder (RD or LI) was driving association signals in the comorbid RD and LI analysis (Fig. 1). We also examined the associations of markers within several previously identified RD and/or LI risk genes, including those recently reported in Luciano *et al.* (2013), in order to present their results with these phenotypic definitions. These genes included: *ABCC13*, *ATP2C2*, *BC0307918*, *CMIP*, *CNTNAP2*, *DAZAP1*, *DCDC2*, *DYX1C1*, *FOXP2*, *KIAA0319*, *KIAA0319L*, *PRKCH*, *ROBO1* and *TDP2*.

Gene-based analyses were performed on each phenotype (comorbid RD and LI, as well as RD and LI individually) using the VEGAS program, similar to the Luciano *et al.* study (Liu *et al.* 2010; Luciano *et al.* 2013). To correct for multiple testing, we set a Bonferroni corrected threshold of *α* = 2.84 × 10^−6^ = 0.05/17 610 genes tested.

### PING replication analyses

Replication analyses were completed in the PING study. Details on the recruitment, ascertainment, neurobehavioral, genetic and neuroimaging methods and data acquisition in the PING study are described in detail elsewhere, but are summarized briefly below (Akshoomoff *et al.* in press, Brown *et al.* 2012; Fjell *et al.* 2012; Walhovd *et al.* 2012). The PING study is a cross-sectional cohort of typically developing children between the ages of 3 and 20 years. Subjects were screened for history of major developmental, psychiatric, and/or neurological disorders, brain injury or medical conditions that affect development. However, subjects were not excluded due to learning disabilities such as RD and LI. The human research protections programs and institutional review boards at the 10 institutions (Weil Cornell Medical College, University of California at Davis, University of Hawaii, Kennedy Krieger Institute, Massachusetts General Hospital, University of California at Los Angeles, University of California at San Diego, University of Massachusetts Medical School, University of Southern California and Yale University) participating in the PING study approved all experimental and consenting procedures. For individuals under 18 years of age, parental informed consent and child assent (for those 7–17 years of age) were obtained. All participants age 18 years and older gave their written informed consent.

Subjects completed the validated study version of the NIH Toolbox Cognition Battery, in which two language- and reading-related tasks were completed: the Oral Reading Recognition Test and Picture Vocabulary Test (Akshoomoff *et al.* in press; Weintraub *et al.* 2013). In the Oral Reading Recognition Test, a word or letter is presented on the computer screen and the participant is asked to read it aloud. Responses are recorded as correct or incorrect by the examiner, who views accepted pronunciations on a separate computer screen. The Picture Vocabulary Test is a measure of receptive vocabulary and administered in a computerized adaptive format. The participant is presented with an auditory recording of a word and four images on the computer screen; the task is to touch the image that most closely represents the meaning of the word.

Subjects were genotyped on the Illumina Human660W-Quad BeadChip (San Diego, CA, USA), with markers used for replication analyses passing quality control filters (sample call rate > 98%, SNP call rate > 95%, minor allele frequency > 5%). We constructed a reference panel as described elsewhere (Brown *et al.* 2012; Fjell *et al.* 2012; Walhovd *et al.* 2012). To assess ancestry and admixture proportions in the PING participants, we used a supervised clustering approach implemented in the admixture software (Alexander *et al.* 2009) and clustered participant data into six clusters corresponding to six major continental populations: African, Central Asian, East Asian, European, Native American and Oceanic. Implementation of ancestry and admixture proportions in the PING subjects is described in detail elsewhere (Brown *et al.* 2012; Fjell *et al.* 2012; Walhovd *et al.* 2012). To prevent possible population stratification, only subjects with a European genetic ancestry factor (GAF) of 1 were included in genetic analysis of behavior. These 440 individuals of European ancestry [mean age of 11.5 (SD = 4.8) years, 53.0% male] were analyzed using quantitative performance on the Oral Reading Recognition and Picture Vocabulary scores with plink v1.07, with age included as a covariate (Purcell *et al.* 2007). To correct for multiple testing (20 total tests = 10 SNPs × 2 language measures), we set statistical thresholds using the false discovery rate with *α* = 0.05 (Benjamini & Hochberg 1995).

### PING imaging analysis

PING imaging techniques, data acquisition and analyses are discussed in depth elsewhere and briefly below (Brown *et al.* 2012; Fjell *et al.* 2012; Walhovd *et al.* 2012). Across the 10 sites and 12 scanners, a standardized multiple modality high-resolution structural MRI protocol was implemented, involving 3D T1- and T2-weighted volumes and a set of diffusion-weighted scans. At the University of California at San Diego, data were obtained on a GE 3T SignaHD× scanner and a 3T Discovery 750× scanner (GE Healthcare, Waukesha, WI, USA) using eight-channel phased array head coils. The protocol included a conventional three-plane localizer, a sagittal 3D inversion recovery spoiled gradient echo T1-weighted volume optimized for maximum gray/white matter contrast (echo time = 3.5 milliseconds, repetition time = 8.1 milliseconds, inversion time = 640 milliseconds, flip angle = 8°, receiver bandwidth = ± 31.25 kHz, FOV = 24 cm, frequency = 256, phase = 192, slice thickness = 1.2 mm), and two axial 2D diffusion tensor imaging (DTI) pepolar scans (30-directions *b* value = 1000, TE = 83 milliseconds, TR = 13 600 milliseconds, frequency = 96, phase = 96, slice thickness = 2.5 mm). Acquisition protocols with pulse sequence parameters identical or near identical to those protocols used at the University of California at San Diego were installed on scanners at the other nine sites. Data were acquired on all scanners to estimate relaxation rates and measure and correct for scanner-specific gradient coil nonlinear warping. Image files in DICOM format were processed with an automated processing stream written in MATLAB (Natick, MA, USA) and C++ by the UCSD Multimodal Imaging Laboratory. T1-weighted structural images were corrected for distortions caused by gradient nonlinearities, coregistered, averaged and rigidly resampled into alignment with an atlas brain. Image postprocessing and analysis were performed using a fully automated set of tools available in the FreeSurfer software suite (http://surfer.nmr.mgh.harvard.edu/) as well as an atlas-based method for delineating and labeling WM fiber tracts (Fischl 2012).

### Diffusion tensor imaging

Diffusion-weighted images were corrected for eddy current distortion using a least square inverse and iterative conjugate gradient descent method to solve for the 12 scaling and translation parameters describing eddy current distortions across the entire diffusion MRI scan, explicitly taking into account the orientations and amplitudes of the diffusion gradient (Zhuang *et al.* 2006). Head motion was corrected by registering each diffusion-weighted image to a corresponding image synthesized from a tensor fit to the data (Hagler *et al.* 2009). Diffusion MRI data were corrected for spatial and intensity distortions caused by B0 magnetic field in-homogeneities using the reversing gradient method (Holland *et al.* 2010). Distortions caused by gradient nonlinearities were corrected by applying a predefined, scanner-specific, nonlinear transformation (Jovicich *et al.* 2006). Diffusion-weighted images were automatically registered to T1-weighted structural images using mutual information (Wells *et al.* 1996) and rigidly resampled into a standard orientation relative to the T1-weighted images with isotropic 2-mm voxels. Cubic interpolation was used for all resampling steps. Conventional DTI methods were used to calculate diffusion measures (Basser *et al.* 1994; Pierpaoli *et al.* 1996). Scanning duration for the DTI sequence was 4:24 min. White matter fiber tracts were labeled using a probabilistic-atlas-based segmentation method (Hagler *et al.* 2009). Voxels containing primarily gray matter or cerebral spinal fluid, identified using FreeSurfer's automated brain segmentation, were excluded from analysis (Fischl *et al.* 2002). Fiber tract volumes were calculated as the number of voxels with probability greater than 0.08, the value that provided optimal correspondence in volume between atlas-derived regions of interest and manually traced fiber tracts.

### Statistical analyses

Imaging-genetics analyses were performed in individuals of European genetic ancestry. Scanner, age, handedness, socioeconomic status and sex were included as covariates in all analyses (Akshoomoff *et al.* in press; Brown *et al.* 2012; Fjell *et al.* 2012; Walhovd *et al.* 2012). 332 subjects of European genetic ancestry had completed imaging measures that passed PING quality control. Fiber tract volumes in 16 tracts of interest were tested by multiple regression analyses in R using the PING data portal (https://mmil-dataportal.ucsd.edu).

## Results

### SNP and gene-based associations

The 10 strongest GWAS associations with comorbid RD and LI in ALSPAC are presented in Table 2. The strongest associations were observed with *ZNF385D* (OR = 1.81, *P* = 5.45 × 10^−7^) and *COL4A2* (OR = 1.71, *P* = 7.59 × 10^−7^) (Table 2). Next, we examined RD and LI individually—with no comorbid cases included—determining whether one disorder was driving these associations. The 10 strongest associations for RD cases and LI cases individually are presented in Tables3 and 4, respectively. The strongest associations with LI were with markers in *NDST4* (OR = 1.83, *P* = 1.40 × 10^−7^) (Table 3). Markers on chromosome 10 (OR = 1.43, *P* = 5.16 × 10^−6^), chromosome 8 (OR = 1.70, *P* = 5.85 × 10^−6^) and the *OPA3* gene (OR = 1.53, *P* = 6.92 × 10^−6^) had the strongest associations with RD (Table 4). Markers with *P* < 0.01 within genes previously implicated in RD and/or LI are presented in Table S1, Supporting Information for each phenotype. The strongest associations with these markers were seen for *KIAA0319* with comorbid RD and LI (rs16889556, *P* = 0.0005177), *FOXP2* with comorbid RD and LI (rs1530680, *P* = 0.0001702), *CNTNAP2* with LI (rs6951437, *P* = 0.0000462) and *DCDC2* with LI (rs793834, *P* = 0.0002679) (Table S1a–S1c). Gene-based analyses were completed on each phenotype (comorbid RD and LI, RD individually and LI individually), and the 10 strongest gene-based associations are presented in Table S2. None of the gene-based associations survived correction for multiple testing; however, the strongest associations were seen with: (1) *OR5H2*, *OR5H6* and *RRAGA* with comorbid RD and LI, (2) *NEK2*, *DLEC1* and *NARS* with LI and (3) *MAP4*, *OR2L8* and *CRYBA4* with RD. Markers with the strongest *P*-values in discovery analyses in *ZNF385D*, *COL4A2* and *NDST4* were carried forward for replication analysis in PING. We observed replication of two markers within *ZNF385D* and performance on the Picture Vocabulary Test (*P* = 0.00245 and 0.004173) (Table 5). However, markers did not replicate with the Oral Reading Recognition Test (*P* > 0.05).

**Table 2:** Associations with comorbid RD and LI cases in ALSPAC (*n* = 174)

Marker	Chr	Base pair	Minor allele	MAF Aff	MAF Unaff	Gene	Odds ratio	*P* value
rs12636438	3	22038281	G	0.3017	0.1927	*ZNF385D*	1.811	5.45 × 10^−7^
rs1679255	3	22022938	C	0.3006	0.1923	*ZNF385D*	1.805	6.87 × 10^−7^
rs9521789	13	109917621	C	0.5201	0.3879	*COL4A2*	1.71	7.59 × 10^−7^
rs1983931	13	109916103	G	0.5201	0.3896	*COL4A2*	1.698	1.06 × 10^−6^
rs9814232	3	21948179	A	0.2931	0.1886	*ZNF385D*	1.784	1.30 × 10^−6^
rs7995158	13	109909718	A	0.5201	0.3911		1.687	1.44 × 10^−6^
rs6573225	14	58354640	C	0.1965	0.1122		1.935	1.56 × 10^−6^
rs4082518	10	17103032	T	0.3103	0.2049	*CUBN*	1.746	2.17 × 10^−6^
rs442555	14	58365937	C	0.1983	0.1149		1.905	2.38 × 10^−6^
rs259521	3	21942154	T	0.2902	0.1885	*ZNF385D*	1.761	2.42 × 10^−6^

Chr, chromosome; MAF Aff, minor allele frequency in affected subjects; MAF Unaff, minor allele frequency in unaffected subjects.

**Table 3:** Associations with LI cases in ALSPAC, excluding comorbid RD cases (*n* = 163)

Marker	Chr	Base Pair	Minor Allele	MAF Aff	MAF Unaff	Gene	Odds Ratio	*P* value
rs482700	4	116286939	G	0.3896	0.2588	*NDST4*	1.827	1.40 × 10^−7^
rs7695228	4	116309516	T	0.3920	0.2636	*NDST4*	1.801	2.94 × 10^−7^
rs1940309	4	116306410	T	0.3865	0.2606	*NDST4*	1.788	4.14 × 10^−7^
rs505277	4	116248257	T	0.3773	0.2528	*NDST4*	1.791	4.35 × 10^−7^
rs476739	4	116248997	A	0.3773	0.2529	*NDST4*	1.79	4.41 × 10^−7^
rs867036	4	116381578	C	0.3957	0.2696	*NDST4*	1.774	5.31 × 10^−7^
rs867035	4	116381423	C	0.3957	0.2697	*NDST4*	1.773	5.45 × 10^−7^
rs2071674	4	2366882	T	0.0920	0.0389	*ZFYVE28*	2.503	1.90 × 10^−6^
rs7694946	4	116413588	C	0.3620	0.2526	*NDST4*	1.678	8.95 × 10^−6^
rs4823324	22	44616787	C	0.2914	0.4143	*ATXN10*	0.581	9.30 × 10^−6^

Chr, chromosome; MAF Aff, minor allele frequency in affected subjects; MAF Unaff, minor allele frequency in unaffected subjects.

**Table 4:** Associations with RD cases in ALSPAC, excluding comorbid LI cases (*n* = 353)

Marker	Chr	Base pair	Minor allele	MAF Aff	MAF Unaff	Gene	Odds ratio	*P* value
rs180950	10	115697957	G	0.456	0.369		1.431	5.16 × 10^−6^
rs2590673	8	126037337	G	0.133	0.083		1.697	5.85 × 10^−6^
rs892100	19	50772522	C	0.228	0.162	*OPA3*	1.526	6.92 × 10^−6^
rs1792745	18	51955991	T	0.187	0.129		1.558	1.22 × 10^−5^
rs12546767	8	126151747	C	0.152	0.099	*KIAA0196*	1.618	1.32 × 10^−5^
rs12634033	3	146524529	C	0.135	0.087		1.646	1.80 × 10^−5^
rs892270	12	105002956	G	0.534	0.451	*NUAK1*	1.395	2.16 × 10^−5^
rs10887149	10	124156994	A	0.278	0.357	*PLEKHA1*	0.690	2.25 × 10^−5^
rs10041417	5	33218502	T	0.226	0.164		1.489	2.58 × 10^−5^
rs6792971	3	68468217	C	0.111	0.068	*FAM19A1*	1.703	2.59 × 10^−5^

Chr, chromosome; MAF Aff, minor allele frequency in affected subjects; MAF Unaff, minor allele frequency in unaffected subjects.

**Table 5:** Replication of associations in PING (*n* = 440)

				Oral Reading Test		Picture Vocabulary Test	
					
Marker	Minor allele	MAF	Gene	Beta	*P* value	Beta	*P* value
rs12636438	G	0.161	*ZNF385D*	−0.1867	0.9452	−2.88	0.004173*
rs1679255	G	0.292	*ZNF385D*	−1.84	0.5016	−3.048	0.002445**
rs9521789	G	0.4370	*COL4A2*	−0.3411	0.7332	0.8647	0.3877
rs476739	A	0.265	*NDST4*	0.5406	0.5891	0.5159	0.6062
rs505277	A	0.280	*NDST4*	0.5406	0.5891	−0.3452	0.7301
rs482700	G	0.278	*NDST4*	0.5498	0.5828	−0.05341	0.9574
rs7695228	A	0.295	*NDST4*	0.6258	0.5318	0.09991	0.9205
rs867036	G	0.378	*NDST4*	0.2605	0.7946	−0.1414	0.8876
rs867035	G	0.377	*NDST4*	0.2961	0.7673	−0.1565	0.8757
rs1940309	A	0.281	*NDST4*	0.6049	0.5456	0.1296	0.8969

MAF, minor allele frequency in full PING sample.

* *P* value less than FDR-adjusted statistical threshold (FDR-adjusted threshold = 0.05 × (2/19) = 0.00526.

** *P* value less than FDR-adjusted statistical threshold (FDR-adjusted threshold = 0.05 × (1/20) = 0.00250.

### Imaging-genetics of *ZNF385D*

**Figure 2:**
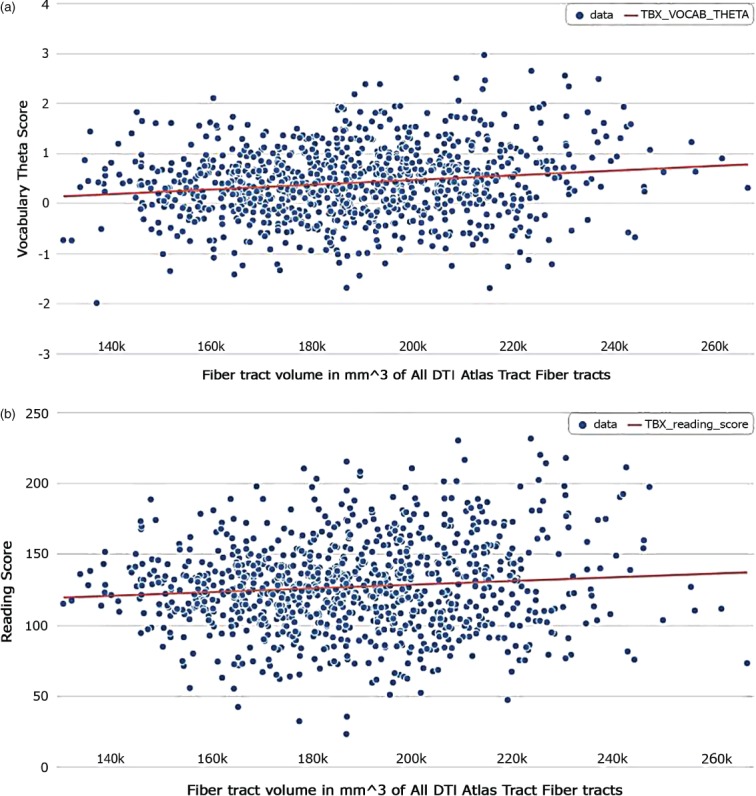
**Association of total fiber tract volumes and neurocognitive tasks.** Relationship of total DTI fiber tract volume with performance on (a) Picture Vocabulary Test and (b) Oral Reading Test. Total DTI fiber tract volumes were predictors of performance on both vocabulary (*P* = 0.000602) and reading (*P* = 0.03596) following correction for age, handedness, gender, scanner device used and socioeconomic status.

To follow-up on the replicated associations of *ZNF385D*, we examined the effects of these variants on fiber tract volumes previously implicated in written and verbal language. Before doing so, we determined that fiber tract volume was a predictor of performance on Oral Reading Recognition and Picture Vocabulary Tests (Fig. 2a,b). Within subjects of only European genetic ancestry, *ZNF385D* genotypes were predictors of overall fiber tract volume as well as fiber tract volumes in the right and left hemispheres (Table 6). *ZNF385D* SNPs were also predictors bilaterally within the inferior longitudinal fasiculus (ILF), inferior fronto-occipto fasiculus (IFO) and temporal superior longitudinal fasiculus (tSLF) in this subset (Table 6). To discern whether these associations between *ZNF385D* and fiber tract volumes reflect global brain volume differences among genotype, we next examined the relationship of *ZNF385D* with both total brain segmentation and total cortical volumes. We found associations for both measures with rs1679255 (*P* = 0.00072 and 0.00027, respectively) and rs12636438 (*P* = 0.000259 and 0.000069, respectively). The effects appeared to be additive in nature, with heterozygous individuals having intermediate phenotypes relative to those homozygous for the major allele and to those homozygous for the minor allele. In fact, when these total brain volume measures were inserted into the model as a covariate, *ZNF385D* associations with DTI fiber tract volumes were no longer present.

**Table 6:** *ZNF385D* associations with DTI fiber tract volumes in subjects with 100% European genetic ancestry (*n* = 332)

	rs1679255		rs12636438	
		
Fiber tract	Slope	*P* value	Slope	*P* value
All	−3329.9	0.044*	−3717.9	0.023*
Right All	−1731.4	0.039*	−1965	0.017*
Left All	−1616.3	0.055	−1775.6	0.033*
Right ILF	−251.3	0.011*	−234.4	0.016*
Left ILF	−256.9	0.0088**	−254.6	0.009**
Right IFO	−200.8	0.032*	−190	0.041*
Left IFO	−221	0.012*	−226.3	0.009**
Right SLF	−168.1	0.06	−206	0.02*
Left SLF	−199.5	0.022*	−212.9	0.013*
Right tSLF	−170.8	0.011*	−180.7	0.0068**
Left tSLF	−163.1	0.023*	−169.9	0.016*
Right pSLF	−153.1	0.079	−182.4	0.034*
Left pSLF	−112.2	0.18	−125.3	0.131
Right SIFC	−148.8	0.052	−165.6	0.029*
Left SIFC	−34.54	0.66	−54.3	0.48
CC	−977.1	0.15	−1181.6	0.081

All, all fiber tracts; CC, corpus callosum; pSLF, parietal superior longitudinal fasiculus; SLF, superior longitudinal fasiculus; SIFC, striatal inferior frontal cortex.

* *P* ≤ 0.05.

** *P* ≤ 0.01.

## Discussion

In this investigation, we sought to identify genes that contribute to the common co-occurrence of RD and LI. In our discovery analyses, we found associations of *ZNF385D* and *COL4A2* in comorbid cases, and of *NDST4* with LI. Next, we observed associations of *ZNF385D* with performance on a vocabulary measure, but not on an oral reading measure, in PING. Association with performance on a vocabulary measure, although not exactly recapitulating the comorbidity phenotype, does provide further evidence for the contribution of *ZNF385D* to language. To gain functional understanding, we interrogated the effects of replicated *ZNF385D* markers on the volumes of language-related fiber tracts. *ZNF385D* markers associated bilaterally with overall fiber tract volumes and overall brain volume.

Studies have shown that RD and LI share genetic contributors (Trzaskowski *et al.* 2013). However, specific genes that contribute to both RD and LI have only recently begun to be examined. These studies have used a candidate gene approach to examine this shared genetic etiology. Such an approach has been successful in showing the shared contribution of *DCDC2*, *KIAA0319*, *FOXP2*, *CNTNAP2*, among others, to both RD and LI (Eicher & Gruen 2013; Graham & Fisher 2013; Newbury *et al.* 2009, 2011; Pinel *et al.* 2012; Rice *et al.* 2009; Scerri *et al.* 2011). In fact, markers within *KIAA0319*, *FOXP2* and *CNTNAP2* (along with *BC0307918*) showed nominal association with comorbid RD and LI in our analyses (*P* < 0.01). RD/LI risk genes also showed a tendency to associate with LI individually (*DCDC2*, *KIAA0319* and *CNTNAP2*) and with RD individually (*CNTNAP2* and *CMIP*) (*P* < 0.01). The lack of replication for other RD/LI risk genes and differences between this study and those of Scerri *et al.* (2011) and Luciano *et al.* (2013) are likely a result of different case definitions and numbers, as we designed our case classifications to capture a wide range of reading- and language-impaired subjects, as opposed to using highly specific neurocognitive measures.

A glaring omission in the genetic investigations of RD and LI is the lack of hypothesis-free methods. These methods allow for discovery of new genes because they do not rely on pre-selected candidates. Here, our GWAS analyses indicate that *ZNF385D* contributes to comorbid RD and LI. Our study is not the first GWAS on reading- and language- related traits. Luciano *et al.* (2013) recently reported a GWAS of quantitative measures of written and verbal language measures in two population-based cohorts, including ALSPAC. They found strong evidence that *ABCC13*, *BC0307918*, *DAZAP1*, among others contribute to performance on these measures, although our analyses did not provide strong evidence for them. The analytical strategies differed in two ways: (1) the use of dichotomous rather than quantitative measures to condition genetic associations and (2) examining reading and language together as opposed to individually. Past association studies of RD and LI have shown differences in results depending on whether associations were conditioned on dichotomous or quantitative phenotypes. For instance, *KIAA0319* tends to associate more readily with quantitative measures, while *DCDC2* associates more often with dichotomized variables (Paracchini *et al.* 2008; Powers *et al.* 2013; Scerri *et al.* 2011). The present study, which examines comorbidity, and that of Luciano *et al.*, which examined performance on reading and language tasks individually, conditioned genetic associations on different traits, which can lead to different statistical associations. Both analytical strategies are valid and have gleaned separate, yet related insight into the genetic underpinnings of written and verbal language. They demonstrate the importance of creative and careful examination of phenotypes when examining neurocognitive and other complex traits.

Following our primary analysis of comorbid RD and LI, we next examined RD and LI individually to determine whether a single disorder was driving the association signals. *ZNF385D* did not associate with either RD or LI individually, indicating that *ZNF385D* contributes to processes related to both RD and LI, as opposed to only one of these disorders. Within PING, we observed associations of *ZNF385D* markers with performance on the Picture Vocabulary Test and not the Oral Reading Recognition Test. Measures of receptive vocabulary (e.g. the Picture Vocabulary Test) are related to both written and verbal language tasks (Scarborough 1990; Wise *et al.* 2007), while performance on decoding measures (e.g. the Oral Reading Recognition Test) appear to be specific to reading. Therefore, the Picture Vocabulary Test may reflect the comorbid RD and LI phenotype used in ALSPAC better than the Oral Reading Recognition Test and explain the association pattern of *ZNF385D* in PING. In addition to *ZNF385D*, we observed suggestive associations of *COL4A2* with comorbid RD/LI and *NDST4* with LI. Neither of these associations replicated in PING, but future studies should attempt to replicate these associations, particularly due to the known involvement of *COL4A2* in porencephaly and white matter lesions (Verbeek *et al.* 2012; Yoneda *et al.* 2012).

Gene-based analyses did not reveal any associations that survived correction for multiple testing. Nonetheless, there were intriguing gene associations that should be investigated in future studies. For instance, with LI, there were suggestive associations with genes on chromosome 19—*IL4I1*, *ATF5*, *NUP62* and *SIGLEC11*—which may correspond to the SLI2 linkage peak (Monaco 2007; SLI Consortium 2002), Luciano *et al.* (2013) found a similar accumulation of suggestively associated genes approximately 5 Mb away from our genes. Additionally, *MAP4*, a microtubule assembly gene, was the strongest associated gene with RD. There is evidence microtubule function plays a key role in reading development as aberrant neuronal migration is thought to contribute to the etiology of RD and other RD candidate genes are thought to interact with microtubules (e.g. *DCDC2* and *ACOT13*) (Cheng *et al.* 2006). Although intriguing, these suggestive findings must be validated in an independent cohort.

The strongest observed associations in this study were with markers within *ZNF385D*. *ZNF385D* has previously been implicated in schizophrenia and attention deficit hyperactivity disorder (ADHD) (Poelmans *et al.* 2011; Xu *et al.* 2013). Both schizophrenia and ADHD are neurobehavioral disorders thought to have core impairments in common with RD and LI, including comprehension and semantic processing (Gilger *et al.* 1992; Li *et al.* 2009; Willcutt *et al.* 2005). Additionally, our observed association of *ZNF385D* on global brain volume may indicate that *ZNF385D* influences various neurocognitive traits through its effect on the entire brain.

There is little known regarding the function of *ZNF385D*, although its zinc finger domain suggests it is a transcriptional regulator. The importance of transcriptional regulation in written and verbal language is not a new concept. The most widely studied language gene, *FOXP2*, is a potent transcription factor that has been shown to regulate another language gene, *CNTNAP2* (Vernes *et al.* 2007; Vernes *et al.* 2011). Additionally, in the DYX2 locus, two risk variants, READ1 within *DCDC2* and the *KIAA0319* risk haplotype, appear to have the capacity to regulate gene expression (Couto *et al.* 2010; Dennis *et al.* 2009; Meng *et al.* 2011) and possibly interact (Ludwig *et al.* 2008; Powers *et al.* 2013), although more evidence is needed to demonstrate these functionalities. *ZNF385D* variants now join this list of putative transcriptional variants that influence written and verbal language skills. The characterization of target genes of *ZNF385D* and of its transcriptional effects on these targets will be an important next step. Additionally, the identification of target genes may generate therapeutic candidates for treatment and remediation of RD and LI. To gain further insight into *ZNF385D*, we performed imaging-genetics analyses of *ZNF385D* and fiber tract volumes of language-related tracts. *ZNF385D* appears to modulate fiber tract and total brain volumes, which may subsequently affect the connectivity and functionality of brain regions important in the efficient, fluent integration of written and verbal language. Thus, identification of target genes and how the modulation of their expression during neural development yields differences in fiber tract and total brain volumes will be vital for dissecting not only the mechanism of *ZNF385D*, but also for the development of core language skills in children.

This study is subject to several limitations. First, although the overall sample size of the ALSPAC is formidable, the number of cases for each definition is relatively small. This is expected in a cross-sectional cohort of the general population as the prevalence of these disorders ranges between 5% and 17% (Pennington & Bishop 2009). The ALSPAC cohort would not be expected to be enriched for RD and/or LI cases. Small sample size could have hindered our statistical power and ability to identify risk genes with small effect size. Second, the reading and language measures performed in the ALSPAC and PING studies were not identical. Phenotypes in PING were treated as a quantitative trait rather than a dichotomous variable as in ALSPAC. Therefore, attempts to replicate associations observed in the ALSPAC cohort may have been hampered as reading/language measures in PING may have captured different skills than those in ALSPAC. However, the associations observed in the PING indicate that *ZNF385D* plays a substantial, consistent role in overall language processes. Third, atlas-derived tract volume measures, like volumes derived from manually traced fiber tracts, are likely underestimates of true fiber volume for most tracts. However, fiber tract volumes were derived consistently for all subjects and likely reflect inter-individual differences. Nonetheless, the strength and independent replication of our associations and the relationship with brain imaging phenotypes strongly implicate *ZNF385D* in core language processes underlying RD and LI.

In conclusion, we identify *ZNF385D* as a novel gene contributing to both RD and LI, as well as fiber tract and overall brain volume. The implication of another transcription factor in communication disorders underscores the importance of transcriptional regulation in neural development of language domains in the brain. Future studies should aim to further characterize the molecular functionality of *ZNF385D* and replicate this association, as well as our nonreplicated associations—*NDST4* and *COL4A2*—in RD, LI and other related disorders.

## upporting Information

**Table S1:** Associations of markers within genes previously implicated in RD and/or LI with (a) Comorbid RD and LI, (b) LI individually and (c) RD individually.

**Table S2:** Gene-based analyses of comorbid RD and LI, LI individually and RD individually. The top 10 gene-based associations for each are shown.
