# Supplementary material for: Genome-wide association study of shared components of reading disability and language impairment
Source: Genes Brain Behav. 2013 Sep 9;12(8):792–801. doi: 10.1111/gbb.12085 (PMC3904347; doi:10.1111/gbb.12085)
Supplement: Supplementary file 1 [file gbb0012-0792-SD1.doc]

Supplemental Table 1: Associations of markers within genes previously implicated in RD and/or LI with (a) Comorbid RD and LI, (b) LI individually, and (c) RD individually.

a) Comorbid RD and LI

| Marker | Gene | Chr. | Base Pair | P-value |
| --- | --- | --- | --- | --- |
| rs16889556 | *KIAA0319* | 6 | 24749584 | 0.0005177 |
| rs1047782 | *TDP2* | 6 | 24758710 | 0.006515 |
| rs1530680 | *FOXP2* | 7 | 114194632 | 0.0001702 |
| rs12667130 | *FOXP2* | 7 | 114213035 | 0.003033 |
| rs6965855 | *CNTNAP2* | 7 | 145348483 | 0.006804 |
| rs985080 | *CNTNAP2* | 7 | 145359118 | 0.006157 |
| rs4726782 | *CNTNAP2* | 7 | 145425012 | 0.005341 |
| rs1718101 | *CNTNAP2* | 7 | 145753721 | 0.0008707 |
| rs10487689 | *CNTNAP2* | 7 | 146835482 | 0.008787 |
| rs1918296 | *CNTNAP2* | 7 | 147655135 | 0.00616 |
| rs737533 | *BC0307918* | 10 | 3353137 | 0.001008 |

b) LI

| Marker | Gene | Chr. | Base Pair | P-value |
| --- | --- | --- | --- | --- |
| rs793845 | *DCDC2* | 6 | 24296970 | 0.005511 |
| rs2799373 | *DCDC2* | 6 | 24303738 | 0.0009664 |
| rs793862 | *DCDC2* | 6 | 24315179 | 0.002443 |
| rs793834 | *DCDC2* | 6 | 24342912 | 0.0002679 |
| rs2792682 | *DCDC2* | 6 | 24380363 | 0.006634 |
| rs807704 | *DCDC2* | 6 | 24408825 | 0.001988 |
| rs707864 | *DCDC2* | 6 | 24413827 | 0.001266 |
| rs12193738 | *KIAA0319* | 6 | 24676372 | 0.00974 |
| rs2817198 | *KIAA0319* | 6 | 24683073 | 0.00559 |
| rs10456309 | *KIAA0319* | 6 | 24697541 | 0.002258 |
| rs985080 | *CNTNAP2* | 7 | 145359118 | 0.006735 |
| rs1554690 | *CNTNAP2* | 7 | 145377266 | 0.006486 |
| rs2533096 | *CNTNAP2* | 7 | 146037312 | 0.004782 |
| rs6951437 | *CNTNAP2* | 7 | 146037340 | 0.0000462 |
| rs344470 | *CNTNAP2* | 7 | 146044430 | 0.001697 |
| rs344468 | *CNTNAP2* | 7 | 146050259 | 0.003965 |

c) RD

| Marker | Gene | Chr. | Base Pair | P-value |
| --- | --- | --- | --- | --- |
| rs4725745 | *CNTNAP2* | 7 | 147032172 | 0.002407 |
| rs12444778 | *CMIP* | 16 | 80330728 | 0.003148 |
| rs1444186 | *CMIP* | 16 | 80330745 | 0.00482 |
