# Supplementary material for: Genome-wide association study of shared components of reading disability and language impairment
Source: Genes Brain Behav. 2013 Sep 9;12(8):792–801. doi: 10.1111/gbb.12085 (PMC3904347; doi:10.1111/gbb.12085)
Supplement: Supplementary file 2 [file gbb0012-0792-SD2.doc]

Supplemental Table 2: Gene-based analyses of comorbid RD and LI, LI individually, and RD individually. The top ten gene-based associations for each are shown.

| Gene | Ch | Start Position | Stop Position | No. SNPS in Gene | p-value |
| --- | --- | --- | --- | --- | --- |
| **RD and LI** |  |  |  |  |  |
| *OR5H2* | 3 | 99484421 | 99485366 | 16 | 0.000072 |
| *OR5H6* | 3 | 99465818 | 99466796 | 19 | 0.000127 |
| *RRAGA* | 9 | 19039371 | 19041021 | 30 | 0.000276 |
| *OR6B3* | 2 | 240633166 | 240634162 | 36 | 0.000294 |
| *UMOD* | 16 | 20251873 | 20271538 | 29 | 0.000307 |
| *A26C1A* | 2 | 131692393 | 131738886 | 1 | 0.000389 |
| *FAM29A* | 9 | 19043140 | 19092902 | 44 | 0.000406 |
| *CHRNA1* | 2 | 175320568 | 175337446 | 23 | 0.000420 |
| *IFIT5* | 10 | 91164418 | 91170733 | 27 | 0.000475 |
| *LOC643905* | 2 | 240629902 | 240631072 | 39 | 0.000562 |
| **LI** |  |  |  |  |  |
| *NEK2* | 1 | 209902744 | 209915590 | 28 | 0.000117 |
| *DLEC1* | 3 | 38055699 | 38139232 | 20 | 0.000171 |
| *NARS* | 18 | 53418891 | 53440175 | 36 | 0.000203 |
| *IL4I1* | 19 | 55084722 | 55124574 | 22 | 0.000305 |
| *PKD2* | 4 | 89147843 | 89217953 | 34 | 0.000313 |
| *ATF5* | 19 | 55123785 | 55129004 | 18 | 0.000344 |
| *NUP62* | 19 | 55101893 | 55124598 | 19 | 0.000402 |
| *SIGLEC11* | 19 | 55144061 | 551556241 | 49 | 0.000578 |
| *ACAN* | 15 | 87147677 | 87219589 | 43 | 0.000633 |
| *PGD* | 1 | 10381671 | 10402788 | 12 | 0.000668 |
| **RD** |  |  |  |  |  |
| *MAP4* | 3 | 47867188 | 48105715 | 18 | 0.000085 |
| *OR2L8* | 1 | 246178782 | 246179721 | 19 | 0.000139 |
| *CRYBA4* | 22 | 25347927 | 25356636 | 40 | 0.000219 |
| *OR2T8* | 1 | 246150942 | 246151881 | 24 | 0.000225 |
| *KIAA1622* | 14 | 93710401 | 93815825 | 42 | 0.000255 |
| *OR2AK2* | 1 | 246195256 | 246196264 | 15 | 0.000315 |
| *DHX30* | 3 | 47819654 | 47866687 | 11 | 0.000316 |
| *GEMIN6* | 2 | 38858830 | 38862610 | 8 | 0.000351 |
| *C20orf10* | 20 | 43435933 | 43440371 | 23 | 0.000450 |
| *PPIF* | 10 | 80777225 | 38862610 | 22 | 0.000493 |
